# Association Between Telehealth Delivery and Same-day Access to Integrated Mental Health in a National VA Sample

**DOI:** 10.1007/s11606-025-09816-9

**Published:** 2025-09-19

**Authors:** Taona P. Haderlein, Darren Lov, Amy Bonilla, Martin L. Lee, Lucinda B. Leung

**Affiliations:** 1 Center for the Study of Healthcare Innovation, Implementation, & Policy, Veterans Affairs Greater Los Angeles Healthcare System, Los Angeles, CA USA; 2 Division of General Internal Medicine and Health Services Research, David Geffen School of Medicine, University of California Los Angeles, Los Angeles, CA USA; 3 Department of Biostatistics, University of California, Los Angeles Fielding School of Public Health, Los Angeles, CA USA

**Keywords:** telehealth, mental health, veterans, primary care

## Abstract

**Background:**

Same-day access to mental health services is associated with better patient outcomes (e.g., diagnosis, treatment). Telehealth appointments via video or phone can improve timely access to care but may complicate in-person care transfers (“warm handoffs”) between primary care and mental health teams.

**Objective:**

To examine associations between receiving telehealth services and same-day access to integrated mental health services within primary care (PCMHI).

**Design, Setting, and Participants:**

This retrospective cohort study included 1,220,902 Veterans who newly initiated PCMHI services between 10/01/18 and 09/30/23.

**Main Measure(s):**

Our primary outcome of interest was whether “same-day access” occurred, defined as a PCMHI visit that took place on the same day as a primary care visit. Our exposure of interest was whether a patient’s initial PCMHI visit took place through in-person versus telehealth, defined as either video or phone. Using multi-level regression models, we examined the association between same-day access and PCMHI visit modality (in-person/phone/video), adjusting for time, region, patient (e.g., demographics, physical and mental health diagnoses), and clinic (e.g., rurality, staffing). Models were stratified by pre-/early-pandemic (FY19-21) versus late-pandemic (FY22-23) periods.

**Results:**

Patients with an initial PCMHI visit conducted through telehealth (video/phone) had 86% lower odds of receiving same-day access than those with an in-person PCMHI visit (95% CI = 0.1444–0.1448). Lower odds of same-day access with PCMHI providers were found for both video (OR = 0.0912; 95% CI = 0.0909–0.0915) and phone (OR = 0.1604, 95% CI = 0.1602–0.1606) visits. Odds of same-day access from primary care to telehealth-based PCMHI care improved with time (OR_FY19-21_ = 0.10, 95% CI = 0.09–0.12; OR_FY22-23_ = 0.18, 95% CI = 0.16–0.20).

**Conclusions and Relevance:**

Results suggest that primary care patients who receive integrated mental health services via telehealth may be less likely to access primary care services on the same day. Further research should consider how traditional primary care workflows (e.g., warm handoffs) may need to adapt to better integrate tele-mental health services.

**Supplementary Information:**

The online version contains supplementary material available at 10.1007/s11606-025-09816-9.

## INTRODUCTION

Ensuring timely care access is integral to effective healthcare delivery for large healthcare systems; yet, it remains challenging for patients seeking mental health services.^1,2^ In response, large healthcare systems such as the Veterans Health Administration (VA) have adopted integrated care models to enable timely mental health access.^3^ Beginning in 2007, primary care mental health integration (PCMHI) providers were integrated into VA primary care clinics nationally to deliver short-term, evidence-based treatment for mild-to-moderate mental health symptoms and to facilitate referrals to specialty mental health and other care when indicated. In 2017, the VA prioritized same-day mental health care access to facilitate rapid initial assessment and treatment planning for new patients, alleviating burden for primary care providers.^4^ Same-day access is associated with increased odds of receiving a mental health diagnosis among newly initiated patients and increased likelihood that such patients receive and attend subsequent mental health appointments.^5^

Offering telehealth appointments via video or phone may be a promising strategy for providing prompt access to mental health services.^6^ Several studies have demonstrated the utility of telehealth for enhancing care coordination, increasing patient engagement, and reducing wait times for various services.^7^ In particular, early in the COVID-19 pandemic, VA video visits partially compensated for the drop in in-person care.^8^ Even as pandemic restrictions subsided and other non-mental health specialties saw a return to pre-pandemic numbers for in-person care, the percentage of mental health visits conducted via telehealth in the VA has remained fairly steady. Despite the demonstrated potential of telehealth for improving VA healthcare access, a handful of studies have raised the concern that telehealth may interfere with the delivery of same-day care.^9–11^ Using data from a large, urban mental health integration clinic, researchers found that patients who received integrated mental health care via telehealth were 46% less likely to have received same-day access than patients who received care in person.^12^ Moreover, patients who received care via telehealth also completed fewer specialty mental health visits during the subsequent six months than patients who received care in person.^13^ Based on these findings, it is also possible that telehealth may negatively affect transitions from primary care to integrated mental health care, reducing the odds of contact with both types of providers on the same day. However, no known studies have examined these patterns on a national scale, nor mitigating factors at the organizational level.

Given VA’s and other healthcare systems’ continued telehealth expansion and ongoing commitment to maximizing mental healthcare access,^14,15^ it is important to understand the impacts of telehealth on same-day access to integrated mental health care for primary care patients. Beginning in 2017, the VA strove to provide same-day mental health and primary care services as part of a larger platform of initiatives targeting suicide prevention among Veterans.^4^ Disruptions to same-day handoffs from primary care to integrated mental health resulting from telehealth use could negatively affect Veterans’ access to care, ultimately worsening mental health outcomes. Furthermore, distinguishing between video and phone visits is important, as there are concerns over equitable access to the Internet and the technology required to conduct video visits^16,17^ for mental health treatment^6,18^ for certain individuals (e.g., seniors, rural Veterans).

This study aimed to examine the association between receiving integrated mental health care via telehealth (video or phone) and the occurrence of same-day access from primary care settings for VA patients nationally. To our knowledge, this is among the largest telehealth studies to examine specific telehealth visit modalities and the timeliness of interdisciplinary care coordination.^19–24^ Based on prior research, we hypothesized that patients who received integrated mental health care via telehealth would be less likely to receive same-day access. Additionally, we examined potential factors at both the patient and the organizational levels that may increase or reduce access to same-day services, as well as patterns of association over time.

## METHODS

### Data Source and Study Sample

We conducted a retrospective longitudinal observational cohort study using electronic health record data from the VA’s Corporate Data Warehouse. Our study cohort included all VA patients who newly initiated PCMHI services between 10/01/18 and 09/30/23 (FY19-23). Specifically, we identified those with PCMHI encounters and no preceding encounter 2 years prior to the study period (FY17-18). This exclusion criteria focuses the study on Veterans who are new to engaging with specialty mental health care and are struggling with getting their mental health care needs met in primary care alone. This study was approved by the VA Greater Los Angeles Healthcare System Institutional Review Board. As all analyses took place with secondary data and no participant recruitment took place, a Waiver of HIPAA Authorization and Waiver of Informed Consent covered all study activities.

### Measures

#### Primary Outcome—Same-Day Access

To achieve *same-day access*, a PCMHI visit must have taken place on the same day as a primary care visit. PCMHI and primary care visits were identified in patient medical records using VA stop codes 534, 527, and 338 (PCMHI) and 123, 124, 317, 322, 323, 348, 350, 372, and 373 (primary care). For each patient’s new initial PCMHI visit, we identified the most recent primary care visit prior to the PCMHI encounter. This binary outcome measure has been described in a prior VA study and is a quality metric monitored by VA’s Office of Mental Health and Suicide Prevention (OMHSP).^13^

#### Exposure Measure—Visit Modality (Telehealth versus In-Person)

Our exposure of interest was whether a patient’s initial PCMHI visit took place through telehealth versus in-person. We defined telehealth visits as a synchronous encounter between a clinician and patient that took place by either video (i.e., secondary stop code 179 or 679) or phone (i.e., primary stop code 527 or 338).

#### Covariates

We adjusted for patient characteristics related to mental health care access. First, we controlled for sociodemographic covariates in our analysis, including patient age, sex, and race/ethnicity. Previous studies found that mental health care access and telehealth use differ by different racial/ethnic groups.^25^ Second, although our sample was defined as patients who had no PCMHI encounters in their VA medical records in the 2 years prior to the study period, we used their medical records to identify whether a patient had pre-existing (prior to PCMHI visit) mental health conditions using the International Classification of Diseases Tenth Revision (ICD-10) codes for (1) posttraumatic stress disorder (PTSD), (2) depressive disorder, and/or (3) substance use disorder (SUD). Diagnostic criteria required one code for any inpatient visit but at least two codes for validation in outpatient visits. Third, we adjusted for a patient’s overall health status using the Elixhauser Comorbidity Index.^26,27^ We used ICD-10 codes to identify patient comorbidities and then categorized each patient as having 0, 1, or 2 + comorbidities.

#### Clinic-level Variables

Timely access to care can also be influenced by clinic resources and organizational capacity to support mental health care integration. First, we controlled for whether clinics were in rural (versus urban) locations or based in community-based outpatient clinics (versus a VA hospital). Second, we included a measure for the primary care teamlet staffing ratio, which we pulled from VA’s Support Service Center Capital Assets (VSSC) Patient Aligned Care Teams Cube.^28^ The VA aims to fully staff each primary care teamlet with at least three supporting members (e.g., nurses, clerks) for every primary care provider (i.e., 3:1 ratio). Based on the low number of clinics meeting this ratio, we dichotomized the variable for teamlet staffing ratio as being at/above versus below 0.5. We also included PCMHI staffing ratios obtained from OMHSP and defined as the ratio of a clinic’s total PCMHI clinician full-time equivalent (FTE)/clinic’s primary care patient population.^29^ We categorized this measure by tertiles within each fiscal year (FY).

### Analysis

First, we ran frequencies and percentages of both patient and clinic characteristics to determine the composition of our study sample. To check for issues with multicollinearity, we also ran a pairwise correlation matrix and checked for VIF tolerance. We then ran bivariate analyses to describe associations between telehealth visits and same-day PCMHI. For our adjusted models, we used multi-level (patient/clinic) logistic regressions to assess associations between our primary predictors (telehealth visit modality) and same-day PCMHI access. In the first model, the exposure variable was any telehealth visit modality (synchronous video *or* phone) versus in-person care for a PCMHI encounter. The second model included video and phone as two separate predictors. We controlled for fixed effects from exogenous temporal trends (by quarter) and for administrative regional differences (by Veterans Integrated Service Network). We used random effect variables to control for clustering at the clinic level.

We also ran two additional analyses. First, to observe if temporal effects were consistent between pre-/early- versus late-COVID-19 pandemic periods, we ran both adjusted models stratified by each period (FY19-21 vs FY22-23). Second, to account for potential racial/ethnic differences in clinic selection, we ran the same two adjusted models with an interaction term between patient race/ethnicity and VA clinic type (hospital-based or community-based clinic). All analyses were performed using R (v. 4.3.1) and significance was determined based on the *p* < 0.05 threshold.

## RESULTS

Our final sample consisted of 1,220,902 Veterans who newly initiated PCMHI services over the 5-year study period (Table 1). About half had an initial PCMHI visit take place over telehealth (44% phone + 11% video). Forty-two percent had a depression diagnosis in their medical record, while 34% and 16% had a diagnosis for PTSD and SUD, respectively. Most were at urban clinics (73%) versus rural clinics (27%). Veterans were split across community-based outpatient clinics (52%) and hospital-based clinics (48%). Patients were split across clinics with primary care teamlet staffing ratios (staff/provider) below and above 0.5 (58% vs 42%, respectively). Almost half (47%) were seen in clinics with a PCMHI staffing ratio at the highest tertile, with 17% in clinics at the lowest tertile. Differences across patients who received same-day access to PCMHI and those who did not were significant for all variables except PTSD diagnosis.

**Table 1:** Characteristics of Study Cohort Across 973 VA Primary Care Clinics (2018–2023)

Characteristic	Overall (*N* = 1,220,902*)	Did not receive same-day access to PCMHI (*N* = 847,120*)	Received same-day access to PCMHI (*N* = 373,782*)
PCMHI visit via telehealth			
No	538,426 (44%)	266,324 (31%)	272,102 (73%)
Yes	682,476 (56%)	580,796 (69%)	101,680 (27%)
PCMHI visit via video			
No	1,081,638 (89%)	721,765 (85%)	359,873 (96%)
Yes	139,264 (11%)	125,355 (15%)	13,909 (3.7%)
PCMHI visit via phone			
No	677,690 (56%)	391,679 (46%)	286,011 (77%)
Yes	543,212 (44%)	455,441 (54%)	87,771 (23%)
PTSD diagnosis			
No	799,827 (66%)	554,857 (66%)	244,970 (66%)
Yes	419,682 (34%)	291,063 (34%)	128,619 (34%)
Unknown	1393	1200	193
Substance use disorder diagnosis			
No	1,025,651 (84%)	713,789 (84%)	311,862 (83%)
Yes	193,858 (16%)	132,131 (16%)	61,727 (17%)
Unknown	1393	1200	193
Depression diagnosis			
No	704,151 (58%)	489,351 (58%)	214,800 (57%)
Yes	515,358 (42%)	356,569 (42%)	158,789 (43%)
Unknown	1393	1200	193
Elixhauser comorbidity			
0 comorbidities	293,961 (24%)	206,291 (24%)	87,670 (23%)
1 comorbidity	314,729 (26%)	212,803 (25%)	101,926 (27%)
2 + comorbidities	610,819 (50%)	426,826 (50%)	183,993 (49%)
Unknown	1393	1200	193
Age category			
18–39	363,641 (30%)	246,767 (29%)	116,874 (31%)
40–64	513,505 (42%)	360,655 (43%)	152,850 (41%)
65–74	222,322 (18%)	156,740 (19%)	65,582 (18%)
75 +	120,036 (9.8%)	81,755 (9.7%)	38,281 (10%)
Unknown	1398	1203	195
Sex			
Female	203,174 (17%)	143,516 (17%)	59,658 (16%)
Male	1,016,335 (83%)	702,404 (83%)	313,931 (84%)
Unknown	1393	1200	193
Race/ethnicity			
Black	273,427 (22%)	191,561 (23%)	81,866 (22%)
Hispanic	135,196 (11%)	89,877 (11%)	45,319 (12%)
Other^‖^	117,440 (9.6%)	80,388 (9.5%)	37,052 (9.9%)
White	693,446 (57%)	484,094 (57%)	209,352 (56%)
Unknown	1393	1200	193
Clinic rurality			
Rural	305,044 (27%)	212,730 (27%)	92,314 (27%)
Urban	823,242 (73%)	576,745 (73%)	246,497 (73%)
Unknown	92,616	57,645	34,971
Clinic type			
Community-based outpatient clinic	637,578 (52%)	422,523 (50%)	215,055 (58%)
Hospital-based clinic	578,263 (48%)	420,495 (50%)	157,768 (42%)
Unknown	5061	4102	959
Primary care teamlet staffing ratio (clinic staff/provider)			
< = 0.5	698,308 (58%)	505,474 (60%)	192,834 (52%)
> = 0.5	514,241 (42%)	336,310 (40%)	177,931 (48%)
Unknown	8353	5336	3017
PCMHI staffing tertile (within fiscal year)			
First tertile	202,423 (17%)	141,561 (17%)	60,862 (17%)
Second tertile	438,204 (37%)	316,658 (38%)	121,546 (33%)
Third tertile	557,146 (47%)	374,516 (45%)	182,630 (50%)
Unknown	23,129	14,385	8744

^*^n (%); mean (SD)

^†^Differences across the two groups were significant for all variables except PTSD diagnosis (based on Pearson’s chi-squared test; Wilcoxon rank sum test)

^‡^Other category included the following groups: Native American/Alaska Native, Native Hawaiian/Pacific Islander, Asian, and those identifying as Multiple Races

*PCMHI*, primary care mental health integration

### Regression Results

In fully adjusted models, patients who newly initiated PCMHI visits via telehealth had lower odds of same-day access. Our first model (Table 2) found that the odds of having same-day PCMHI services in primary care were lower when those visits took place through telehealth (video *or* phone) as compared to in-person (OR = 0.1446, 95% CI = [0.1444, 0.1448]). Our second model (Appendix Table 1) showed both video and phone visits were associated with lower odds of same-day access than in-person visits (video: OR = 0.0912, CI = [0.0909, 0.0915])/phone: OR = 0.1604, CI = [0.1602, 0.1606]).

**Table 2:** Adjusted Odds of Receiving Same-Day Access to Primary Care Mental Health Integration (PCMHI) Services

	**Odds ratio**	**95% CI**^§^
PCMHI visit modality (ref: in-person)		
Telehealth (phone or video)	0.1446	[0.1444, 0.1448]
PTSD	1.055	[1.048, 1.063]
Substance use disorder	1.06	[1.05, 1.07]
Depression	1.025	[1.019, 1.031]
Elixhauser comorbidity (ref: 0 comorbidities)		
1 comorbidity	1.18	[1.17, 1.19]
2 + comorbidities	1.068	[1.061, 1.075]
Age (ref: < 40)		
40–64	0.86	[0.85, 0.87]
65–74	0.82	[0.81, 0.83]
75 +	0.86	[0.85, 0.87]
Sex (ref: female)		
Male	1.008	[1.002, 1.014]
Race/ethnicity		
Black	1.05	[1.04, 1.06]
Hispanic	1.09	[1.07, 1.10]
Other^‖^	1.09	[1.07, 1.12]
Clinic rurality		
Urban	0.91	[0.90, 0.92]
Clinic type (ref: community-based outpatient clinic)		
Hospital-based clinic	1.25	[1.00, 1.57]
Primary care teamlet staffing ratio (clinic staff/provider) (ref: < = 0.5)		
> 0.5	1.069	[1.065, 1.072]
PCMHI staffing tertile within FY (ref: first tertile)		
Second tertile	1.149	[1.145, 1.153]
Third tertile	1.344	[1.339, 1.350]

^§^Findings were significant at the < 0.05 *p*-value for all variables except for clinic type (*p* = 0.054)

‖Other category included the following groups: Native American/Alaska Native, Native Hawaiian/Pacific Islander, Asian, and those identifying as Multiple Races

^¶^We controlled for fixed effects from exogenous temporal trends (by quarter) and for administrative regional differences (by VA regional network). We used random effect variables to control for clustering at the clinic level

*PCMHI*, Primary Care Mental Health Integration

Both models indicated slightly higher odds of receiving same-day access to PCMHI visits for patients with pre-existing mental health diagnoses: PTSD (OR = 1.055, CI = [1.048, 1.063]), substance use disorder (OR = 1.06, 95% CI = [1.05, 1.07]), and depression (OR = 1.025, CI = [1.019, 1.031]). Veterans with worse overall health, as measured by the Elixhauser score, were also more likely to receive same-day access to PCMHI than Veterans with a 0 score (1 comorbidity, OR = 1.18, CI = [1.17, 1.19]/2 + comorbidities, OR = 1.068, CI = [1.061, 1.075]).

Veterans in older age groups had lower odds of receiving same-day access to PCMHI compared to Veterans less than 40 years old (40–64, OR = 0.86, CI = [0.85, 0.87]/65–74, OR = 0.82, CI = [0.81, 0.83]/75 +, OR = 0.86, CI = [0.85, 0.87]). We found that among racial/ethnic groups, Black Veterans (OR = 1.05, CI = [1.04, 1.06]) and Hispanic Veterans (OR = 1.09, CI = [1.07, 1.10]) were more likely to receive same-day access to PCMHI compared to White Veterans.

Clinic-level covariates (other than rural location) were positively associated with same-day PCMHI services. Receiving care at a hospital-based clinic was associated with higher odds of receiving same-day access compared to community-based clinics (OR = 1.25, CI = [1.00, 1.57]). Clinics with teamlet staffing ratios above 0.5 were more likely to have patients receiving same-day PCMHI access (OR = 1.069, CI = [1.065, 1.072]), as were clinics with PCMHI staffing ratios in the highest tertiles within each fiscal year (OR = 1.344, CI = [1.339, 1.350]).

### Additional Analyses

First, stratified by time period (Table 3), we found that patients who had their initial PCMHI visit via telehealth were more likely to receive same-day access late-pandemic (OR = 0.18, CI = [0.16, 0.20]) compared to pre-/early-pandemic (OR = 0.10, CI = [0.09, 0.12]) periods (Appendix Table 2 for results broken out by phone and video). Second, in our interaction model assessing racial/ethnic disparities for same-day PCMHI access across VA clinic type, we found results similar to the main models above.

**Table 3:** COVID Sensitivity Analysis—Adjusted Odds of Receiving Same-Day Access to Primary Care Mental Health Integration (PCMHI) Services

	*Fiscal year (FY) 2019–2021*		*FY 2022–2023*	
	**Odds ratio**	**95% CI**	**Odds ratio**	**95% CI**
PCMHI visit modality (ref: in-person)				
Telehealth (phone or video)	0.10	[0.09, 0.12]	0.18	[0.16, 0.20]
PTSD	1.03	[1.01, 1.06]	1.11	[1.07, 1.14]
Substance use disorder	1.06	[1.03, 1.10]	1.07	[1.04, 1.11]
Depression	1.06	[1.03, 1.09]	0.98	[0.95, 1.02]
Elixhauser comorbidity (ref: 0 comorbidities)				
1 comorbidity	1.15	[1.10, 1.20]	1.24	[1.19, 1.28]
2 + comorbidities	1.02	[0.97, 1.07]	1.16	[1.11, 1.21]
Age (ref: < 40)				
40–64	0.88	[0.86, 0.90]	0.84	[0.82, 0.86]
65–74	0.84	[0.81, 0.87]	0.79	[0.76, 0.83]
75 +	0.92	[0.88, 0.96]	0.80	[0.76, 0.84]
Sex (ref: female)				
Male	1.01	[0.96, 1.05]	1.01	[0.97, 1.05]
Race/ethnicity				
Black	1.04	[1.02, 1.07]	1.07	[1.04, 1.09]
Hispanic	1.08	[1.05, 1.11]	1.09	[1.05, 1.13]
Other**‖**	1.10	[1.06, 1.13]	1.09	[1.05, 1.13]
Clinic rurality				
Urban	0.97	[0.95, 0.99]	0.84	[0.82, 0.87]
Clinic type (ref: community-based outpatient clinic)				
Hospital-based clinic	1.33	[1.15, 1.55]	1.17	[0.99, 1.38]
Primary care teamlet staffing ratio (clinic staff/provider) (ref: < = 0.5)				
> 0.5	1.00	[0.91, 1.09]	1.09	[1.01, 1.18]
PCMHI staffing tertile (ref: first tertile)				
Second tertile	1.02	[0.86, 1.21]	1.07	[0.89, 1.29]
Third tertile	1.21	[0.98, 1.50]	1.39	[0.98, 1.97]

^§^Findings were significant at the < 0.05 *p*-value for all variables except for clinic type (*p* = 0.054)

‖Other category included the following groups: Native American/Alaska Native, Native Hawaiian/Pacific Islander, Asian, and those identifying as Multiple Races

^¶^We controlled for fixed effects from exogenous temporal trends (by quarter) and for administrative regional differences (by VA regional network). We used random effect variables to control for clustering at the clinic level

*PCMHI*, Primary Care Mental Health Integration

## DISCUSSION

Same-day access has been linked with greater engagement in specialty mental health treatment and prioritized as part of VA suicide prevention efforts.^4,5,13,30^ However, VA patients whose initial PCMHI visit was conducted through telehealth (video or phone) were less likely to have received same-day access from primary care than those whose PCMHI visit took place in person. Although patient needs and readiness for mental health care may vary, this negative association persisted even after accounting for patient and clinic-level characteristics. There is thus concern that telehealth may interfere with a key mechanism designed to improve mental health outcomes among Veterans: prompt referral to integrated mental health providers from primary care. Alternatively, patients who initiate care via telehealth may experience different needs and readiness to engage in mental health treatment, highlighting the importance of adapting referral pathways to align with telehealth patients’ preferences and circumstances. Although telehealth is an effective modality for increasing overall mental health access,^31–33^ our findings support the need for strategies to support care coordination among patients whose integrated mental health services are being delivered remotely.

Our findings are in line with past smaller-scale studies conducted in single healthcare systems reporting lower odds of same-day integrated mental health services in primary care for patients who receive telehealth versus in-person care.^12,13^ They also contribute new findings at the national scale suggesting that telehealth might introduce care coordination challenges between mental health and primary care specialties, as well as introduce mutable clinic-level factors (e.g., staffing) for improvement.^29,34,35^ More research is needed to identify specific mechanisms, including patient and provider behaviors, that may account for lower odds of same-day access to PCMHI for telehealth-based visits, such as the availability of warm handoffs between both specialties in different locations. Provider preferences may play a critical role in options presented to patients, as not all may feel comfortable seeing patients, especially new patients, through telehealth.^36^ Likewise, some patients may prefer to be seen in person, while others may find it easier scheduling virtual appointments that work more easily within their schedules. Future research studies should also consider examining how telehealth affects the likelihood of patient engagement with mental health services over time.

Large healthcare systems should pursue measures to mitigate potential decrements to same-day access associated with telehealth use. While patient-level variables help adjust for traditional barriers to care and preferences for how to receive care, organizational level variables account for facility resources and barriers to offering PCMHI services. Consistent with prior research, our study found that PCMHI staffing was positively associated with same-day access, suggesting that availability of PCMHI providers to receive primary care referrals may facilitate referral completion.^29,37^ Similarly, the PACT teamlet staffing ratio indicated that higher primary care staffing levels facilitating referrals to PCMHI were associated with higher odds of same-day access.^34,35^ While adequate primary care and mental health staffing may help improve same-day access to PCMHI, workflows that support virtual care referrals likely also facilitate same-day access for new mental health patients identified in primary care. For example, some VA mental health integration clinics utilize virtual warm handoffs, where multiple providers can join the same video call with a patient from different workstations.^38^ Other existing technologies, such as instant messaging between providers and/or support staff, can also be leveraged to facilitate prompt referrals for telehealth patients.^39^ User-friendly scheduling platforms and sufficient trainings for clinic support staff should also be considered when assessing potential enablers to same-day telehealth appointments.^40^

### Limitations

Our study has some limitations. First, electronic health record and administrative data can be subject to potential miscoding of phone and video visits,^41^ similarly affecting observational telehealth research broadly. Nonetheless, we were reassured by observations of similar associations between pre-/early- and late-pandemic periods, the latter when telehealth coding was known to have improved. Second, we were unable to account for patient or provider preferences regarding visit modality and appointment times. For example, providers may only offer telehealth visits to certain non-acute patients for safety concerns, or patients may be unable to fit in the additional appointment that same day. Future research can consider collecting information on scheduling options presented to patients and how that affects same-day access. Third, although we controlled for historical mental health diagnoses, we could not adjust for patient-level symptom severity at the index primary care visit or readiness to engage in mental health treatment. These factors likely affected patient decision-making around whether and when to engage in PCMHI. Fourth, we only capture short-term, same-day PCMHI attendance, as this is used to measure PCMHI’s ability to receive new primary care patients. A longer-term analysis would enable examination of longitudinal trends around mental health engagement.

## CONCLUSIONS

The VA has broadly expanded telehealth across all clinics nationally, and our study points out a positive trend in PCMHI access for telehealth patients over time, suggesting that VA primary care teams are successfully refining processes to support prompt mental health referrals for remote patients. However, same-day access remained worse for telehealth patients relative to in-person. More research is needed to understand whether this trend is due to patient or provider preferences or larger organizational obstacles specific to telehealth practice such as scheduling availability or other workflow challenges. Adequate staffing in primary care and mental health were identified as protective factors for same-day access in this study. These and other related strategies (e.g., modify existing clinic workflows to accommodate telehealth patients, leverage existing technologies for care coordination) could be considered to optimize primary care transitions to mental health care for Veterans who receive telehealth services.

## Supplementary Information

Below is the link to the electronic supplementary material.

Supplementary Material 1 (DOCX 25.0 KB)
