# Supplementary material for: Association Between Telehealth Delivery and Same-day Access to Integrated Mental Health in a National VA Sample
Source: J Gen Intern Med. 2025 Sep 19;41(5):1214–21. doi: 10.1007/s11606-025-09816-9 (PMC13083669; doi:10.1007/s11606-025-09816-9)
Supplement: Supplementary file 1 — Supplementary Material 1 (DOCX 25.0 KB) [file 11606_2025_9816_MOESM1_ESM.docx]

**Appendix Table 1. Adjusted Odds of Receiving Same-Day Primary Care Mental Health Integration (PCMHI) Services (Video and Phone Visits Broken Out)**

|  | Odds Ratio | 95% CI^§^ |
| --- | --- | --- |
| PCMHI Visit Modality (ref: in-person) |  |  |
| Phone Visit | 0.1604 | [0.1602, 0.1606] |
| Video Visit | 0.0912 | [0.0909, 0.0915] |
| PTSD | 1.056 | [1.049, 1.064] |
| Substance Use Disorder | 1.059 | [1.049, 1.068] |
| Depression | 1.027 | [1.021, 1.033] |
| Elixhauser Comorbidity  (ref: 0 comorbidities) |  |  |
| 1 Comorbidity | 1.18 | [1.17, 1.19] |
| 2+ Comorbidities | 1.067 | [1.060, 1.073] |
| Age (ref: <40) |  |  |
| 40-64 | 0.85 | [0.84, 0.86] |
| 65-74 | 0.80 | [0.79, 0.81] |
| 75+ | 0.84 | [0.83, 0.85] |
| Sex (ref: Female) |  |  |
| Male | 1.002 | [0.996, 1.008] |
| Race/Ethnicity |  |  |
| Black | 1.05 | [1.04, 1.07] |
| Hispanic | 1.09 | [1.07, 1.11] |
| Other^‖^ | 1.10 | [1.07, 1.13] |
| Clinic Rurality |  |  |
| Urban | 0.907 | [0.900, 0.914] |
| Clinic Type  (ref: Community-Based Outpatient Clinic) |  |  |
| Hospital-Based Clinic | 1.24 | [0.98, 1.55] |
| Primary Care Teamlet Staffing Ratio  (clinic staff/provider) (ref: <=0.5) | | |
| >0.5 | 1.061 | [1.057, 1.064] |
| PCMHI Staffing Tertile within FY  (ref: First Tertile) |  |  |
| Second Tertile | 1.160 | [1.156, 1.163] |
| Third Tertile | 1.351 | [1.346, 1.357] |
| § Findings were significant at the <.05 p-value for all variables except for sex (p=0.502) and clinic type (p=0.068)  ‖ Other category included the following groups: Native American/Alaska Native, Native Hawaiian/Pacific Islander, Asian, and those identifying as Multiple Races.  ¶We controlled for fixed effects from exogenous temporal trends (by quarter) and for administrative regional differences (by VA regional network). We used random effect variables to control for clustering at the clinic level.  PCMHI = Primary Care Mental Health Integration | | |

**Appendix Table 2. COVID Sensitivity Analysis - Adjusted Odds of Receiving Same-Day Primary Care Mental Health Integration (PCMHI) Services (Video and Phone Visits Broken Out)**

|  | *2019-2021* | | *2022-2023* | |
| --- | --- | --- | --- | --- |
|  | **Odds Ratio** | **95% CI** | **Odds Ratio** | **95% CI**^§^ |
| PCMHI Visit Modality (ref: in-person) |  |  |  |  |
| Phone Visit | 0.10 | [0.09, 0.12] | 0.23 | [0.20, 0.27] |
| Video Visit | 0.09 | [0.07, 0.11] | 0.08 | [0.07, 0.10] |
| PTSD | 1.03 | [1.01, 1.06] | 1.11 | [1.08, 1.14] |
| Substance Use Disorder | 1.06 | [1.03, 1.09] | 1.06 | [1.03, 1.10] |
| Depression | 1.06 | [1.03, 1.09] | 0.99 | [0.96, 1.02] |
| Elixhauser Comorbidity (ref: 0 comorbidities) |  |  |  |  |
| 1 Comorbidity | 1.15 | [1.10, 1.20] | 1.24 | [1.20, 1.29] |
| 2+ Comorbidites | 1.02 | [0.97, 1.07] | 1.16 | [1.11, 1.21] |
| Age |  |  |  |  |
| 40-64 | 0.88 | [0.85, 0.90] | 0.82 | [0.80, 0.84] |
| 65-74 | 0.83 | [0.80, 0.86] | 0.75 | [0.72, 0.79] |
| 75+ | 0.91 | [0.87, 0.95] | 0.76 | [0.72, 0.80] |
| Sex (ref: Female) | |  |  |  |
| Male | 1.00 | [0.96, 1.05] | 0.99 | [0.96, 1.03] |
| Race/Ethnicity | | | | |
| Black | 1.04 | [1.02, 1.07] | 1.07 | [1.04, 1.10] |
| Hispanic | 1.08 | [1.05, 1.11] | 1.09 | [1.06, 1.13] |
| Other‖ | 1.10 | [1.06, 1.13] | 1.09 | [1.05, 1.13] |
| Clinic Rurality |  |  |  |  |
| Urban | 0.97 | [0.95, 0.99] | 0.84 | [0.82, 0.87] |
| Clinic Type (ref: Community-Based Outpatient Clinic) | | | | |
| Hospital-Based Clinic | 1.33 | [1.15, 1.55] | 1.14 | [0.97, 1.35] |
| Primary Care Teamlet Staffing Ratio (clinic staff/provider)  (ref: <=0.5) | | |  |  |
| >0.5 | 1.00 | [0.91, 1.09] | 1.08 | [1.00, 1.17] |
| PCMHI Staffing Tertile (ref: First Tertile) | | | | |
| Second Tertile | 1.03 | [0.87, 1.22] | 1.05 | [0.87, 1.26] |
| Third Tertile | 1.22 | [0.98, 1.51] | 1.36 | [0.97, 1.90] |
| § Findings were significant at the <.05 p-value for all variables except for gender, 2+ comorbidities (Elixhauser Index), teamlet staffing ratio, and PCMHI staffing tertile pre-Covid, and gender, depression diagnosis, facility type, and PCMHI staffing tertile post-Covid.  ‖ Other category included the following groups: Native American/Alaska Native, Native Hawaiian/Pacific Islander, Asian, and those identifying as Multiple Races.  ¶We controlled for fixed effects from exogenous temporal trends (by quarter) and for administrative regional differences (by VA regional network). We used random effect variables to control for clustering at the clinic level.  PCMHI = Primary Care Mental Health Integration | | | | |
